# 응급실 이동식 X-ray 검사 시 자외선 차단 커튼의 방사선 안전 효과 평가: 비교실험연구

**DOI:** 10.7586/jkbns.25.027

**Published:** 2025-08-14

**Authors:** Mihyun Han, Nam-Ju Lee, Kyeonghwan Jeong, Dongkyung Jung

**Affiliations:** 1 Department of Nursing, Keimyung College University, Daegu, Korea; 1 계명문화대학교 간호학과; 2 College of Nursing·The Research Institute of Nursing Science, Seoul National University, Seoul, Korea; 2 서울대학교 간호대학·서울대학교 간호과학연구소; 3 Department of Radiography, Daegu Health College, Daegu, Korea; 3 대구보건대학교 방사선학과

**Keywords:** Radiation protection, Scattering, radiation, Portable X-ray, Emergency nursing, Patient safety, 방사선방어, 산란선, 이동식 X-ray 의료검사, 응급간호, 환자안전

## Abstract

**Purpose:**

This study evaluated the radiation shielding effectiveness of general hospital curtains and ultraviolet (UV)-blocking curtains in emergency departments by measuring the spatial distribution of scatter-radiation dose rates during portable X-ray examinations.

**Methods:**

Four shielding conditions were assessed: no curtain, a general hospital curtain, a 99.9% UV-blocking curtain (V2), and a combination of two UV-blocking curtains (V2 & V7). Spatial dose rates were measured at six locations surrounding a simulated patient bed: Feet 0°, Right 0°, Horizontal 45°, Feet 0° & Vertical 30°, Right 0° & Vertical 30°, and Horizontal 45° & Vertical 30°. All measurement points were set 150 cm from the phantom center to simulate the distance between the patients and the surrounding beds.

**Results:**

At a distance of 150 cm horizontally from the phantom, the spatial dose rate was lower when a 99.9% UV-blocking curtain was used than when a general hospital curtain or no curtain was used. Among all curtain types, the V2 & V7 99.9% UV-blocking curtains demonstrated the highest shielding efficiency. The lowest spatial dose rate was observed at Feet 0°, where a bed tray was present, while the highest was at Right 0° & Vertical 30°.

**Conclusion:**

In beds equipped with the V2 & V7 99.9% UV-blocking curtains and tray, radiation exposure can be minimized by positioning personnel at the foot of the patient. These findings support the use of UV-blocking curtains as a convenient shielding method during portable X-ray procedures in emergency departments. The findings may also guide caregivers and patients to safer positions during examinations.

## 서론

### 1. 연구의 필요성

응급의료기관은 외상이나 급성질환의 환자에게 신속한 의학적 처치를 제공하여 생명의 존속이 유지되도록 하는 의료시설이다. 응급환자의 진료와 처치를 위해서는 의학적 진단을 위한 의료 영상 검사가 필수적이다. 그러나 응급 상황이거나 환자의 거동이 불편한 경우에는 환자의 영상 검사를 위해 영상의학과로 이동하는 것이 힘들다. 따라서 진단을 위한 검사를 지체할 수 없는 경우 이동식 X-ray 의료검사 시스템(portable X-ray system)을 이용하여 응급실 내에서 의료 영상을 획득하는 경우가 많다[1]. 이동식 X-ray 검사는 응급환자 진료 시 신속한 영상진단을 가능하게 하지만, 좁은 공간에 다수의 환자와 의료진이 밀집되어 있는 응급실에서는 주변 인원에 대한 X-ray 산란선의 피폭 위험을 증가시킨다[2]. 더욱이 주변에 차폐체를 설치하지 않고 검사하는 경우가 많으므로 응급실에서 환자를 처치하고 있는 간호사, 주변의 환자와 보호자 등에게 X-ray 산란선이 피폭될 수 있다[3].

질병관리청은 1977년 국제 방사선 방호위원회(International Commission on Radiological Protection, ICRP)의 방사선 방호원칙(As Low As Reasonably Achievable) 권고에 따라 방사선 피폭을 최소화하기 위해 의료기관의 방사선 종사자에게 피폭 관리를 하고 있다[4]. 대표적으로 열형광선량계(thermoluminescence dosimeter), 형광유리선량계(photoluminescence glass dosimeter) 및 광자극발광선량계(optically stimulated luminescence dosimeter)를 근무 중에 항상 착용하여 분기별로 측정하며[5], 매년 교육 및 건강검진을 통해 관리하고 있다[6]. 또한, ICRP는 방사선 작업종사자와 일반인의 방사선 선량한도를 권고하고 있으며, 간호사는 일반인의 선량한도 권고사항인 연간 1 mSv(의료 및 자연방사선 제외) 이하가 기준이다[7].

간호사가 필수적으로 근무하는 수술실 의료진의 위치에는 상대적으로 높은 공간선량의 분포도가 나타났으며[8], 수술실 내 모든 공간에서 산란선이 측정되어 근무자의 방호와 교육의 필요성을 보고하였다[9]. 그러나 응급실 간호사의 방사선 피폭에 대한 연구는 부족한 실정이다. 응급실 간호사가 환자에게 간호 수행을 하는 공간에서 이동식 X-ray 의료검사를 시행하는 경우가 많아 의료방사선에 피폭될 가능성이 높으므로[1], 방사선 방어 및 산업재해 방지를 위한 간호 관리 방안이 필요하다.

방사선 방어를 위해 사용되는 납치마 및 납커튼의 연구는 이루어지고 있지만[10,11], 경제적 문제와 물리적 무게 등의 납의 특성으로 인해 사용자의 불편한 점이 나타난다[12]. 이동식 X-ray 의료검사 시 납차폐 적용 여부나 환자로부터의 거리와 각도에 따른 공간선량률을 측정한 연구들[13-15]은 있으나, 응급실에서 사용 가능한 간편한 차폐체의 효과를 실험적으로 분석한 연구는 제한적이었다. 현재 대부분의 응급실에서는 사생활 보호와 공간 구획을 위해 커튼이 사용되고 있으나, 해당 커튼의 방사선 차폐 효과는 공식적으로 검증되지 않은 경우가 많다. 특히, 자외선 차단 커튼 등 다양한 종류의 커튼이 일반적으로 판매되고 있음에도 불구하고, 그 차폐 효율성에 대한 과학적 근거가 부족하다. 자외선은 X-ray와 가장 근접한 전자기파로 X-ray 다음 순으로 파장이 길고 에너지가 낮다[16]. 또한 멸균에 자외선, X-ray, 감마선이 사용되므로 유사한 성질을 가진 전자기파이다[17,18]. 그러므로 자외선 99.9% 차단 커튼이 X-ray의 차폐에 효과가 있을 것으로 추정하여 가설을 설정하였다.

따라서 본 연구에서는 간호사 및 환자, 보호자의 방사선 피폭의 안전을 위해 검사 환자를 기준으로 X-ray 산란선(scatter-radiation)을 측정하고, 방사선 피폭 방어를 위해 쉽게 사용할 수 있는 응급실 커튼 및 자외선 차단 커튼의 유용성을 평가하여 응급실에서 편리하게 이용할 수 있는 차폐체 사용을 제안하고자 한다. 본 연구를 통해 X-ray 산란선 차폐의 효율성이 높은 커튼을 권고하여 응급의료기관에 종사하는 간호사와 응급실 내원 환자와 보호자의 방사선 피폭 방호를 위한 기초 자료를 제시하고자 한다.

### 2. 연구의 목적

연구의 구체적인 목적은 다음과 같다.

첫째, 커튼 적용 조건(무차폐, 일반 커튼, 단일 자외선 99.9% 차단 커튼, 이중 자외선 99.9% 차단 커튼)에 따른 X-ray 산란선의 차폐 효과를 비교한다.

둘째, 환자로부터의 거리와 각도에 따른 측정 위치에 따라 X-ray 산란선의 차폐 효과를 비교한다.

### 3. 연구 가설

본 연구에서는 다음과 같은 가설을 설정하였다.

가설 1: 커튼의 적용 조건에 따라 X-ray 산란선의 차폐 효과에 유의한 차이가 있을 것이다.

가설 2: 측정 위치에 따라 X-ray 산란선의 차폐 효과에 유의한 차이가 있을 것이다.

## 연구 방법

### 1. 연구 설계

본 연구는 간호사와 환자, 보호자의 방사선 피폭에 대한 안전을 평가하기 위해 의료방사선을 이용하여 커튼 적용 조건에 따라 X-ray 산란선의 차폐 효과를 확인하는 비교실험연구이다.

### 2. 연구 대상

본 연구는 이동식 X-ray 의료검사 시스템에서 발생되는 X-ray 산란선의 공간선량률에 대한 분포도를 측정하며, 응급실 내 일반 커튼과 자외선 차단 커튼에 의한 산란선의 차폐 효과를 측정하고자 한다. 이에 따라 X-ray를 조사받는 인체모형 팬텀과 X-ray 산란선이 발생되는 공간, 차폐의 효과를 확인하기 위해 적용하는 커튼이 연구 대상이다.

### 3. 연구 도구

#### 1) 실험 장비

##### (1) 이동식 X-ray 장비

이동식 X-ray 의료검사 장비(GM85, Samsung, Suwon, Gyeonggi-do, Korea)를 이용하여 진단 참고수준 가이드라인[19] 및 응급실에 사용하고 있는 성인 여성 흉부 X-ray 바로 누운 자세 (anteroposterior supine) 검사 조사 조건 75 kVp, 320 mA, 10 ms, field size 17×17 inch, Source to image receptor distance 100 cm에서 조사하였다.

##### (2) 팬텀

방사선 흡수 및 Hounsfield unit 값이 인체와 비슷한 전신 팬텀(PBU-50, Kyoto Kagaku, Kyoto, Japan)을 이용하였다. 팬텀의 키는 165 cm, 무게는 50 kg이며 물질은 연조직(urethane based resin: density 1.06), 합성뼈(epoxy resin: density 1.31) 및 머리뼈(epoxy resin: density 1.11)로 구성되어 있다.

##### (3) 커튼(차폐체) 적용 조건

산란선 차폐 효과를 확인하기 위해 일반적으로 구매하기 쉬운 커튼을 사용하였다. 본 실험에는 무차폐(no curtain), 응급실 내 일반 커튼(N8802, Curtain4u Co, Seongnam, Gyeonggi-do, Korea), 단일 자외선 99.9% 차단 커튼(V2 blackout-curtain, Hitex Global Co, Namyangju, Gyeonggi-do, Korea), 자외선 99.9% 차단 이중 커튼(V2 blackout-curtain & V7 blackout-curtain, Hitex Global Co, Namyangju, Gyeonggi-do, Korea)을 사용하였다. FITI 시험연구원(Daegu, Korea)의 자외선(ultraviolet, UV) 차단율 검사를 통해 자외선(UV-A, UV-B, UV-R) 차단율 99.9%를 확인하였다.

##### (4) 방사선 측정기

공간선량률은 비례계수관 서베이미터(FH 40G-L, Thermo Scientific Co, Tokyo, Japan)를 사용하여 측정하였다. 서베이미터는 2024년 1월 30일에 검교정을 완료한 것을 사용하였다(교정인자 평균 1.39; 1000 uSv 범위, 교정인자 평균 1.43, 측정불확도 7.5%; 신뢰수준 약 95%).

#### 2) 실험 환경

**Figure 1.**
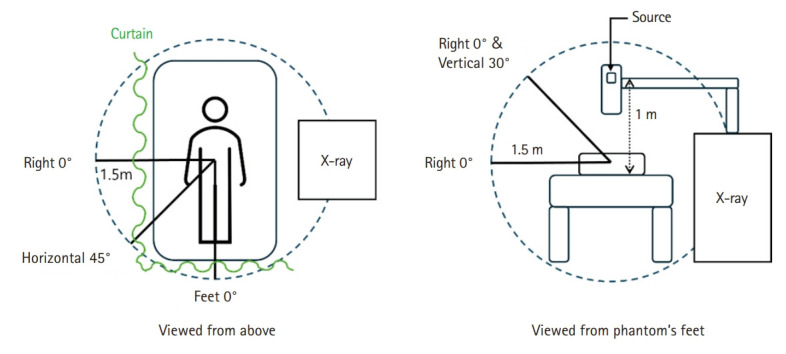
Schematic of the location of the spatial dose rate survey system, viewed from above and from the phantom’s feet.

**Figure 2.**
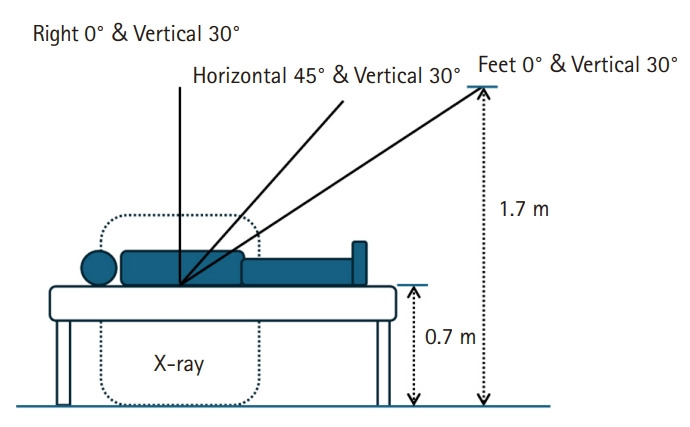
Schematic of the location of the spatial dose rate survey system, viewed from the right side.

응급실 내 환경을 구현하기 위하여 대구가톨릭대학교 칠곡가톨릭병원에서 실험하였다. 응급의료에 관한 법률 시행규칙[20]에 따른 권역응급의료센터 응급환자 진료구역 병상 간 간격 기준에 따라 침대 간 거리를 재현하였다. 침대 크기는 90 cm (depth) × 200 cm (width) × 70 cm (height)이었으며, 발판(foot board)에 위치한 식판은 침대 형태로 유지하고 환자의 체위는 응급환자 흉부 촬영 기준에 따라 바로 누운 자세를 가정한 상황에서 측정을 진행하였다. 공간선량률의 측정 거리는 응급의료에 관한 법률 시행규칙[20]의 권역응급의료센터의 지정 기준 중 병상 간 간격이 150 cm 이상을 확보할 것으로 규정되어 있는 것을 근거로, 보호자 또는 간호사가 이동식 X-ray 의료검사 장비로 검사 시 주로 대기하는 공간 및 침대 간 거리 등을 고려하여 150 cm(환자 중심에서 측정거리)으로 결정하였다. 측정 위치는 이동식 X-ray 의료검사 장비가 환자의 왼쪽에 위치하였을 때 보호자 또는 간호사가 주로 대기하게 공간으로 Figure 1과 같이 각도는 발쪽(Feet 0°), 오른쪽(Right 0°), 대각선(Horizontal 45°)으로 하였다. 또한 Figure 2와 같이 Figure 1 각도에서 높이 170 cm으로 30° 수직 위(Feet 0° & Vertical 30°, Right 0° & Vertical 30°, Horizontal 45° & Vertical 30°) 측정하였다. 이는 방사선이 조사되었을 때 보호자 또는 간호사에게 방사선 생물학적 미치는 영향이 높은 생식기(0°에서 측정)와 갑상선과 안구(30°에서 측정)의 공간선량률을 측정하기 위함이다.

전 세계 일반인 자연방사선 피폭선량 평균은 2.4 mSv/y이며, 국내는 3.2 mSv/y (최대 300 nSv/h)로 나타난다[21]. 이에 따라 본 연구는 자연방사선 300 nSv/h (약 200 ± 100 nSv/h) 이하의 자연방사선(background)에서 차폐체 커튼의 유무 및 자외선 차단 성능에 따라 공간선량률을 비교 분석하였다.

### 4. 자료 수집

자료수집에 앞서 연구 설계의 검증을 위해 의료방사선 전문가인 방사선학과 교수 3인(평균 경력 15년 이상)에게 자문하여 수정•보완하였다. 실험 기간은 2024년 8월 6일부터 8월 13일까지였다. X-ray 조사는 자격을 가진 방사선사를 섭외하여 시행하였으며, 실험 조건의 신뢰도 확보를 위하여 자문가 3인 중 방사선학과 교수 2인과 함께 커튼 종류 등이 변경될 때마다 조사 조건 및 측정 위치를 확인하였다. 또한, X-ray 조사 전 자연방사선을 측정한 후 실험 방법에 따른 조사 조건으로 1분 간격으로 각 10회씩 반복 측정을 시행하여 평균값으로 분석하였다.

### 5. 자료 분석

수집된 자료는 SPSS version 24.0 (IBM Corp., Armonk, NY, USA)을 이용하여 분석하였다. 각 조건에 의해 측정된 산란선(공간선량률)은 평균과 표준편차로 분석하였고, Shapiro-Wilk 방법을 이용하여 정규성을 검정하였다. 커튼(차폐체) 유무와 측정 위치에 따른 산란선(공간선량률)의 차이는 one-way ANOVA로 분석하였고 사후검정은 Scheffé test를 사용하였다. 모든 경우의 통계적 유의수준은 *p* < .05를 기준으로 하였다.

### 6. 윤리적 고려

본 연구의 실험 대상은 무생물이므로 연구윤리심의위원회의 승인을 받지는 않았다. 실험 당시 X-ray 조사 피해를 방어하기 위하여 공간 내 모든 실험자는 전신 및 갑상선 차폐복(lead apron and thyroid shield)을 착용하였으며, 피폭선량을 측정하는 개인피폭 열형광선량계를 착용하였다.

## 연구 결과

### 1. 커튼 적용 조건에 따른 공간선량률 비교 분석 (가설 1 검증)

측정 위치 6곳에 따라 무차폐, 응급실 내 일반 커튼(N8802), 단일 자외선 99.9% 차단 커튼(V2) 및 이중 자외선 99.9% 차단 커튼(V2 & V7)을 적용하여 비교하였다(Table 1). 모든 위치에서 이중 자외선 99.9% 차단 커튼(V2 & V7)을 적용했을 때 공간선량률이 가장 낮게 측정되었다. Right 0°에서는 자외선 99.9% 차단 커튼 V2와 V2 & V7의 차이가 유의미하게 나타나지 않았으나, 다른 위치에서는 이중 자외선 99.9% 차단 커튼(V2 & V7)이 V2보다 유의미하게 더 낮은 공간선량률을 보였다 (*p* < .001). Feet 0° & Vertical 30°와 Right 0° & Vertical 30°에서는 응급실 내 일반 커튼(N8802)이 있을 때보다 커튼이 없을 때(무차폐) 공간선량률이 유의미하게 낮게 확인되었으나(*p* < .001), 다른 위치에서는 유의미한 차이가 확인되지 않았다.

### 2. 측정 위치에 따른 공간선량률 비교 (가설 2 검증)

커튼 네 가지 종류를 적용하여 측정 위치 6곳의 공간선량률을 비교하였다(Table 2). 모든 커튼 적용 상황에서 Feet 0°가 가장 낮게 공간선량률이 측정되었으며, 다음으로 Horizontal 45°가 확인되었으며, Right 0° & Vertical 30°에서 가장 높게 공간선량률이 측정되었다(*p* < .001).

**Figure 3.**
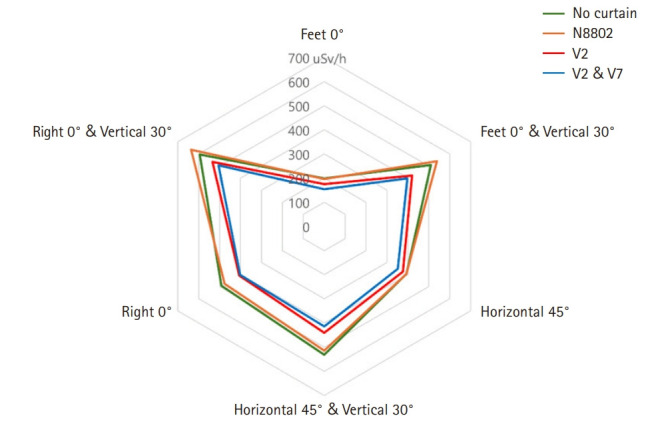
Spatial dose rates according to curtain type and measurement location.

위 2가지 결과를 종합하여 Figure 3으로 나타내었다. 커튼의 종류에 상관없이 Feet 0°의 공간선량률이 가장 낮으며, 위치에 상관없이 이중 자외선 99.9% 차단 커튼(V2 & V7)을 적용하는 것이 공간선량률이 가장 낮은 것을 확인할 수 있다.

## 논의

본 연구는 응급실에서 자주 수행되는 이동식 X-ray 의료검사 시스템의 X-ray 조사 시 차폐체 적용 조건과 측정 위치에 따라 X-ray 산란선의 차폐 효과를 비교하여, 방사선 피폭 방호를 위한 기초 자료를 제공하기 위해 수행하였다. 차폐체로는 응급실에서 사생활 보호 및 공간 구획을 위해 일반적으로 사용되는 커튼을 선정하였다. 측정 위치별로 무차폐, 응급실 내 일반 커튼, 단일 자외선 99.9% 차단 커튼과 이중 자외선 99.9% 차단 커튼을 적용하여 그 차폐 효과를 비교하였다. 특히, 자외선 99.9% 차단 커튼의 X-ray 산란선 차폐 효과를 실증적으로 검토한 점에서 본 연구는 의의가 있다.

커튼 적용 조건에 따른 연구 결과, 단일 자외선 99.9% 차단 커튼(V2)과 이중 자외선 99.9% 차단 커튼(V2 & V7)을 사용할 경우, 무차폐 또는 일반 커튼 대비 통계적으로 유의미하게 낮은 공간선량률이 확인되었다. 이는 자외선 차단 기능을 갖춘 커튼이 자외선과 파장이 유사한 X-ray 산란선에 대해서도 일정 수준의 차폐 효과를 가질 수 있음을 시사한다. 일반인은 자연방사선으로 연간 약 2.4 mSv를 피폭받고 있으며, 인공방사선의 경우 연간 유효선량 한도는 1 mSv로 규정되어 있다[5]. 또한 방사선 발생장치를 일시적으로 사용할 경우 일반인에 대한 선량은 연간 선량한도를 초과하지 아니하는 범위 내에서 시간당 20 uSv까지 허용된다[22]. 본 실험에서 확인된 무차폐 상태와 이중 자외선 99.9% 차단 커튼 적용 간 X-ray 산란선의 공간선량률 차이의 평균은 82.53 uSv/h로, 법적 허용 기준 대비 약 4배 이상 차폐 효과를 나타냈다. 이는 자외선 차단 커튼이 일반인의 인공방사선 피폭량을 실질적으로 감소시키는 효과가 있음을 보여준다.

커튼이 없을 때와 일반 커튼을 적용했을 때는 위치에 따라 유의미한 차이가 나타나지 않거나, Feet 0° & Vertical 30°와 Right 0°& Vertical 30°에서는 커튼이 없을 때가 더 낮게 나타나는 것을 확인할 수 있었다. 이는 자연방사선(background radiation)의 영향으로 볼 수 있으며 일반 커튼은 자연방사선과 비슷한 수치의 산란선도 차폐하지 못한다는 것을 확인할 수 있는 결과이다. 자연방사선은 우주선, 라돈, 지구에 존재하는 방사성 동위원소 물질(우라늄, 토륨 등)에서 방출하는 방사선이다[16]. 즉 자연에서 빛을 방출하기 때문에 자연방사선이라고 하며, 토양, 음식, 태양 등에서 방출되고 인간이 통제할 수 없어 피폭을 측정하지 않는다. 따라서 인공방사선의 피폭을 최소화하는 것이 중요하다.

환자로부터의 거리와 각도에 따라 설정된 측정 위치 중, 커튼 적용 조건과 상관없이 Feet 0°에서 가장 낮은 공간선량률이 나타났다. 이는 침대의 식판이 물리적 차폐 역할을 한 것으로 보인다. Feet 0°에서 납차폐(lead plate)를 적용한 Chiang 등[13]의 연구에서는 가장 낮은 공간선량률이 확인되었고, 발판 방향에 식판이 없는 침대에서 측정한 Brady 등[14]의 연구에서는 가장 높은 공간선량률이 확인되었기 때문이다. 그러므로 이동식 X-ray 의료검사 수행 시 간호사나 보호자가 환자에게서 멀리 떨어질 수 없다면 침대의 식판으로 차폐의 효과를 볼 수 있는 환자의 발판 방향에 위치하는 것이 방사선 피폭을 최소화할 수 있음을 기억해야 한다. 그러나 Feet 0° & Vertical 30°의 공간선량률이 낮은 것은 아니므로 Feet 0°에 위치한 생식기는 보호가 되지만 Vertical 30°에 위치한 방사선 감수성이 높은 갑상선이나 안구는 보호하지 못한다고 볼 수 있어 유의가 필요하다.

또한, Horizontal 45°와 Horizontal 45° & Vertical 30°가 Right 0°와 Right 0° & Vertical 30°보다 공간선량률이 낮은 것을 확인하였다. 이러한 현상은 방사선발생장치에서 환자 방향으로 방사선을 조사하기 위한 양극 각도에 의해 경사효과(heel effect)가 작용하여[16], 환자의 발(양극측)과 가까운 Horizontal 45°보다 환자의 머리(음극측)와 가까운 Right 0°에서 방사선량이 높게 나타나는 것으로 판단된다. 실험 당시 침대의 위치와 공간의 문제로 환자의 머리를 양극측으로 설정할 수 없었기 때문에 이러한 결과가 나타난 것으로 보이며, 추후에는 본 연구와 반대로 적용하였을 때에도 경사효과가 나타나서 공간선량률이 반대로 측정되는지 검증할 필요가 있다.

본 연구 결과에 따르면, 환자 중심으로부터 150 cm의 거리에서는 차폐를 적용하지 않았을 때 X-ray 산란선의 영향이 있었다. 산란선은 거리역자승의 법칙(distance inverse square law)에 따라 방사선량이 거리의 제곱에 반비례하므로[16], 150 cm 보다 더 먼 곳에서 이동식 X-ray 검사가 종료될 때까지 대기하는 것이 권장된다. 실제로 납차폐 없이 검사를 시행하는 경우에는 200 cm 이상 떨어져 있어야 산란선의 영향을 줄일 수 있다는 연구 결과들도 보고되고 있다[13,15]. 그러나 본 연구에서는 거리에 따른 공간선량률의 변화를 측정하지 못했다는 제한점이 있다. 따라서 국내 응급실과 유사한 환경이며 차폐가 없는 상황에서 이동식 X-ray 의료검사 시행 시 방사선 피폭을 최소화할 수 있는 안전 거리에 대한 후속 연구가 필요하다.

다수의 간호사와 보호자 등이 이동식 X-ray 검사를 진행하는 동안 방사선 피폭을 고려하지 않고 불분명한 위치와 거리에서 대기하는 경우가 많다. 본 연구 결과를 통해 응급실 내에서 이동식 X-ray 의료검사 시 간호사 본인의 안전을 위한 침상에서의 거리와 위치를 인식하고 적용할 수 있다. 또한 주변 환자와 보호자들에게도 150 cm의 거리는 피폭의 영향력이 있으므로 안전하지 않으며, 거리역자승의 법칙에 따라 거리를 두고 서 있어야 한다는 것을 교육할 수 있는 기초간호학적 근거를 마련하였다. 환자의 위급 상태에 따라 150 cm 이내에 위치해 있어야 할 경우에는 환자 발판 방향의 식판을 차폐체로 사용하여 발 아래쪽에서 대기하는 것이 피폭을 최소화할 수 있다는 것을 기억하고 적용해야 한다. 또한 응급실 등 이동식 X-ray 의료검사를 수행하는 장소에서는 커튼 구입 시 자외선 99.9% 차단 커튼을 구매하여 방사선 차폐체로 활용할 것을 권고한다. 본 연구는 기초간호학적 영역에서 방사선 안전에 대한 실천적 지식과 간호중재의 근거를 제시함에 의의가 있다. 다만, 본 연구에서 확인한 위치와 자외선 99.9% 차단 커튼을 적용하는 것이 X-ray 피폭의 영향에서 완전히 벗어나는 것은 아니므로 간호사들의 경각심 상승을 위한 교육과 피폭 차단에 대한 추가 연구들이 필요하다.

## 결론

본 연구는 응급실에서 자주 수행되는 이동식 X-ray 의료검사 시스템을 사용한 영상의학적 검사에 의한 피폭선량을 수치화하여 공간선량분포에 대한 자료를 제공하였다. 또한, 가장 편리하게 접근할 수 있는 방호 대책인 사생활 보호를 위한 커튼의 차폐율을 확인하였다. 커튼이 없거나 일반 커튼을 적용한 상황보다 단일 99.9% 자외선 차단 커튼을 적용했을 때 공간선량률이 더 낮게 측정되었으며, 특히 이중 99.9% 자외선 차단 커튼 적용 시 가장 공간선량률이 낮았다. 위치로는 침대의 식판이 위치한 Feet 0°이 공간선량률이 가장 낮았다. 반면에 Right 0° & Vertical 30°에서 공간선량률이 가장 높았다. 따라서 이중 99.9% 자외선 차단 커튼을 적용하고 환자의 발판 방향에 위치하는 것이 피폭을 가장 피할 수 있다. 본 연구를 통해 응급실에서 이동식 X-ray 검사 시 방사선으로부터 간호사 스스로의 안전을 지키며, 환자와 보호자의 대기 위치에 대해 숙지하고 안내할 수 있는 근거 자료를 확보하였다. 응급실 내의 피폭 방호 대책을 마련할 수 있는 기초 자료로 사용되기를 바란다.

## Figures and Tables

**Table 1.** Comparison of Spatial Dose Rates by Curtain Type

Location	Curtain type	M ± SD (μSv/h)	F	*p*	Scheffé test
Feet 0°	No curtain^a^	199.40 ± 10.65	49.40	< .001	d < c < a,b
	N8802 general hospital curtain^b^	196.80 ± 10.35			
	V2 99.9% UV-blocking curtain^c^	174.50 ± 7.81			
	V2 & V7 99.9% UV-blocking curtains^d^	153.90 ± 9.33			
Feet 0° & Vertical 30°	No curtain^a^	509.50 ± 11.39	221.46	< .001	d < c < a < b
	N8802 general hospital curtain^b^	539.70 ± 15.49			
	V2 99.9% UV-blocking curtain^c^	420.90 ± 13.49			
	V2 & V7 99.9% UV-blocking curtains^d^	397.60 ± 17.12			
Horizontal 45°	No curtain^a^	392.60 ± 12.38	23.94	< .001	d < c <b
	N8802 general hospital curtain^b^	393.10 ± 17.26			d < a
	V2 99.9% UV-blocking curtain^c^	376.30 ± 9.98			
	V2 & V7 99.9% UV-blocking curtains^d^	352.10 ± 8.35			
Horizontal 45° & Vertical 30°	No curtain^a^	531.40 ± 14.34	168.68	< .001	d < c < a,b
	N8802 general hospital curtain^b^	514.30 ± 16.20			
	V2 99.9% UV-blocking curtain^c^	440.50 ± 10.50			
	V2 & V7 99.9% UV-blocking curtains^d^	414.90 ± 13.22			
Right 0°	No curtain^a^	492.20 ± 12.44	121.86	< .001	c,d < a,b
	N8802 general hospital curtain^b^	476.00 ± 14.84			
	V2 99.9% UV-blocking curtain^c^	406.10 ± 11.36			
	V2 & V7 99.9% UV-blocking curtains^d^	401.60 ± 14.70			
Right 0° & Vertical 30°	No curtain^a^	595.80 ± 12.37	173.51	< .001	d < c < a < b
	N8802 general hospital curtain^b^	635.90 ± 18.91			
	V2 99.9% UV-blocking curtain^c^	534.70 ± 14.66			
	V2 & V7 99.9% UV-blocking curtains^d^	505.60 ± 8.53			

M = Mean; SD = Standard deviation; UV = Ultraviolet.

**Table 2.** Comparison of Spatial Dose Rates by Measurement Location

Curtain type	Location	M ± SD (μSv/h)	F	*p*	Scheffé test
No curtain	Feet 0°^a^	199.40 ± 10.65	1308.66	< .001	a < c < b,e < d < f
	Feet 0° & Vertical 30°^b^	509.50 ± 11.39			
	Horizontal 45°^c^	392.60 ± 12.38			
	Horizontal 45° & Vertical 30°^d^	531.40 ± 14.34			
	Right 0°^e^	492.20 ± 12.44			
	Right 0° & Vertical 30°^f^	595.80 ± 12.37			
N8802 general hospital curtain	Feet 0°^a^	196.80 ± 10.35	923.15	< .001	a < c < e < d < b < f
	Feet 0° & Vertical 30°^b^	539.70 ± 15.49			
	Horizontal 45°^c^	393.10 ± 17.26			
	Horizontal 45° & Vertical 30°^d^	514.30 ± 16.20			
	Right 0°^e^	476.00 ± 14.84			
	Right 0° & Vertical 30°^f^	635.90 ± 18.91			
V2 99.9% UV-blocking curtain	Feet 0°^a^	174.50 ± 7.81	1074.16	< .001	a < c < b,e < d < f
	Feet 0° & Vertical 30°^b^	420.90 ± 13.49			
	Horizontal 45°^c^	376.30 ± 9.98			
	Horizontal 45° & Vertical 30°^d^	440.50 ± 10.50			
	Right 0°^e^	406.10 ± 11.36			
	Right 0° & Vertical 30°^f^	534.70 ± 14.66			
V2 & V7 99.9% UV-blocking curtains	Feet 0°^a^	153.90 ± 9.33	908.92	< .001	a < c < b,d,e < f
	Feet 0° & Vertical 30°^b^	397.60 ± 17.12			
	Horizontal 45°^c^	352.10 ± 8.35			
	Horizontal 45° & Vertical 30°^d^	414.90 ± 13.22			
	Right 0°^e^	401.60 ± 14.70			
	Right 0° & Vertical 30°^f^	505.60 ± 8.53			

M = Mean; SD = Standard deviation; UV = Ultraviolet.

## Data Availability

Please contact the corresponding author for data availability.
